# Renal Dysfunction, Coronary Artery Lesion Complexity, and Adverse Cardiovascular Outcomes in Patients With Acute Coronary Syndrome

**DOI:** 10.31083/RCM38389

**Published:** 2025-07-23

**Authors:** Qiang Chen, Yike Li, Siqi Yang, Haoming He, Yingying Xie, Wei Wang, Long Wang, Yanxiang Gao, Lin Cai, Shiqiang Xiong, Jingang Zheng

**Affiliations:** ^1^Institute of Clinical Medical Sciences, China-Japan Friendship Hospital, Chinese Academy of Medical Sciences, Peking Union Medical College, 100029 Beijing, China; ^2^Department of Cardiology, China-Japan Friendship Hospital, 100029 Beijing, China; ^3^Department of Cardiology, The Third People's Hospital of Chengdu, Affiliated Hospital of Southwest Jiaotong University, 610031 Chengdu, Sichuan, China

**Keywords:** SYNTAX score, estimating glomerular filtration rate, cystatin C, acute coronary syndrome, mediation

## Abstract

**Background:**

Renal dysfunction is linked to both the complexity of coronary artery lesions and the prognosis of acute coronary syndrome (ACS). However, the nature of this intricate relationship remains unclear. Therefore, this study aimed to investigate the mechanisms through which coronary lesion complexity mediates the association between renal dysfunction and adverse cardiovascular outcomes in patients with ACS.

**Methods:**

This analysis included 1400 ACS patients who underwent percutaneous coronary intervention (PCI). Renal function was assessed using the estimated glomerular filtration rate (eGFR), calculated according to the 2021 Chronic Kidney Disease Epidemiology Collaboration (CKD-EPI) equation, which incorporates both creatinine and cystatin C. Coronary lesion complexity was evaluated using the baseline SYNTAX score (bSS). The associations among eGFR, bSS, and major adverse cardiovascular events (MACEs) were examined using survival analysis, restricted cubic spline (RCS) analysis, and mediation analysis.

**Results:**

A total of 229 MACEs (16.4%) occurred over a median follow-up of 31.03 (27.34, 35.06) months, including 99 all-cause deaths (7.0%), 41 myocardial infarctions (2.9%), and 123 unplanned revascularizations (8.9%). After multivariate adjustment, both the eGFR and bSS significantly predicted MACEs across the total population and various subgroups. Mediation analysis showed that bSS mediated 16.49%, 14.76%, 12.87%, and 11.95% of the correlation between eGFR and MACEs in different adjusted models.

**Conclusion:**

The relationship between renal dysfunction and MACEs in ACS patients is partially mediated by coronary lesion complexity. This finding underscores the importance of integrating kidney function assessments with coronary anatomical evaluations to develop individualized risk stratification strategies.

## 1. Introduction

Acute coronary syndrome (ACS) is a severe manifestation of coronary artery disease, characterized by increased morbidity and mortality [1]. Renal dysfunction is recognized as a significant risk factor in the progression and prognosis of atherosclerotic coronary vessel disease (ASCVD) [2, 3, 4, 5]. In the United States, the prevalence of chronic kidney disease (CKD) among patients undergoing percutaneous coronary intervention (PCI) is 30.5% for those with ST-elevation myocardial infarction (STEMI) and 42.9% for those with non-ST-elevation myocardial infarction (NSTEMI) [6]. Several studies have demonstrated that as the severity of renal impairment increases, the incidence of cardiovascular death and myocardial infarction significantly increases [7, 8, 9].

CKD leads to alterations in calcium and phosphorus metabolism [10], which, along with traditional metabolic risk factors such as diabetes mellitus, inflammation, and disorders of lipid metabolism, collectively accelerate the progression of atherosclerotic lesions [5, 11, 12, 13, 14]. Consequently, patients with CKD frequently exhibit complex and severe coronary artery lesions, characterized by multivessel disease and arterial calcification [15, 16, 17]. Among dialysis patients, the prevalence of coronary artery calcification ranges from 25% to 68.3% [18, 19, 20], with an annual progression rate between 20.8% and 27.6% [21, 22]. Previous studies have demonstrated that impaired renal function serves as an independent prognostic factor for baseline SYNTAX scores (bSS) [23]—which is a validated metric for determining the complexity of coronary artery disease [16, 17]. This complexity complicates revascularization procedures and may lead to adverse outcomes after PCI, including mortality and repeat revascularization in ACS.

In 2023, the American Heart Association (AHA) proposed a novel framework termed the cardiovascular-kidney-metabolic syndrome (CKM) to highlight the interconnections among metabolic risk factors, renal dysfunction and the cardiovascular system [13, 24]. However, there is currently a lack of studies exploring the pathway between CKD and coronary lesion complexity in the prognosis of ACS. Therefore, this study aimed to explore the role of the bSS in the relationship between estimated glomerular filtration rate (eGFR), calculated using the 2021 Chronic Kidney Disease Epidemiology Collaboration (CKD-EPI) equation incorporating both creatinine and cystatin C [25, 26], and adverse cardiovascular events in ACS patients.

## 2. Methods

### 2.1 Study Population

A cohort of 1400 patients from the Third People’s Hospital of Chengdu, Sichuan, China, who underwent PCI between July 2018 and December 2020, were included in the study. Plasma creatinine and cystatin C measurements were recorded for each participant. The study excluded patients with: (1) prior coronary artery bypass grafting (CABG) surgery; (2) structural heart disease necessitating surgical or percutaneous treatment; (3) severe hepatic/respiratory dysfunction; (4) advanced malignancies with limited life expectancy; (5) in-hospital mortality; and (6) more than 10% of critical medical data missing.

### 2.2 Data Collection

Demographic characteristics, medical background, and key clinical parameters were extracted from electronic health records. Fasting venous blood samples were analyzed for lipid profiles, fasting blood glucose, homocysteine, renal biomarkers (serum creatinine, cystatin C), brain natriuretic peptide (BNP), and cardiac troponin T (cTnT) using standardized assays. The bSS was computed using an online calculator available at https://syntaxscore.org/. Preprocedural angiograms were independently assessed by two blinded evaluators, with discrepancies resolved by a third arbitrator. All data were meticulously entered into a specialized computer database, which was then rigorously checked for quality to ensure precision and reliability. eGFR values were calculated using the 2021 CKD-EPI equation, which integrates both creatinine and cystatin C measurements. This method yields a more accurate estimation of the glomerular filtration rate (GFR) than equations relying solely on creatinine [25].

### 2.3 Follow-up 

Follow-up assessments were conducted at 1, 3, 6, and 12 months post-discharge, followed by annual evaluations via telephone interviews or clinic visits. Trained personnel prospectively documented clinical events throughout the follow-up period. The primary endpoint was major adverse cardiovascular events (MACEs), defined as a composite of all-cause mortality, nonfatal myocardial infarction (MI), or ischemia-driven unplanned revascularization. Secondary endpoints included all-cause mortality, cardiac death, MI, unplanned revascularization and nonfatal stroke. All endpoints underwent rigorous adjudication by an independent committee based on standardized criteria, including review of medical records and imaging data.

### 2.4 Sample Size Calculation

The sample size was calculated based on the expected incidence of MACEs over two years (16%), with a target power of 0.8, a two-sided significance level (α = 0.05), and an assumed hazard ratio (HR) of 1.2 per 10-unit decrease in eGFR. The standard deviation (SD) of eGFR was assumed to be 15 units, and a 5% loss to follow-up was accounted for. Using a Cox proportional hazards regression model for continuous variables, the required sample size was calculated to be approximately 700 participants.

### 2.5 Statistical Analysis

Continuous variables were presented as mean ±SD or median (interquartile range [IQR]) based on distributional normality. Group comparisons were performed using appropriate statistical methods based on the data distribution. Categorical variables were reported as counts (percentages) and analyzed using χ^2^ tests or Fisher’s exact tests as appropriate. Missing data were addressed using multiple imputation with fully conditional specification to preserve statistical validity and mitigate potential estimation bias. This calculation ensures adequate statistical power to detect the specified effect size of eGFR on MACE risk. To better illustrate the association between kidney function decline and adverse cardiovascular outcomes, we transformed eGFR values by taking the negative and dividing by 10 for inclusion as continuous variables in analyses.

The correlation between the bSS and eGFR was explored using Spearman’s correlation analysis. Logistic regression analysis was adopted to analyze the association between risk factors including eGFR and the angiographic severity of coronary artery disease (CAD) (bSS ≥22). The dose-response association between eGFR, bSS, and adverse outcomes in ACS patients were analyzed using restricted cubic spline (RCS). Patients were divided into two groups based on predefined thresholds for eGFR (<60 vs. ≥60 mL/min/1.73 m^2^) and bSS (<22 vs. ≥22), and subsequently into four groups combining eGFR and bSS to observe differences in outcome events. Cumulative event rates were estimated using Kaplan-Meier curves, with between-group differences assessed via log-rank tests. Time-to-event Cox regression models were used to evaluate associations between eGFR, bSS, and cardiovascular outcomes. In order to assess the consistency of the predictive utility of eGFR and bSS across diverse patient subgroups, we conducted predefined analyses stratified by age (≤65 vs. >65 years), sex (male vs. female), body mass index (BMI) (<24 vs. ≥24 kg/m^2^), hypertension, diabetes mellitus, acute myocardial infarction (AMI) status, and smoking history (all categorized as yes/no). Interaction terms were incorporated into multivariable Cox regression to evaluate potential effect modifications between these variables and primary outcomes, thereby strengthening the interpretability of the findings.

Mediation analysis was performed using the ‘mediation’ package in the R Programming Language 4.4.1 (R Foundation for Statistical Computing, Vienna, Austria). The analysis evaluated the mediating role of coronary artery lesion complexity (bSS as a continuous variable) in the association between renal dysfunction (eGFR as a continuous variable) and MACEs (time-to-event endpoint), adjusted for multiple covariates in separate multivariable Cox regression models. Bayesian methods were applied for the estimation of mediation effects. A significant mediating effect was established when meeting all three criteria: (a) statistically significant indirect effect (95% credible interval excluding null); (b) significant total effect (*p* < 0.05 in Cox regression); (c) positive proportion-mediated estimate (>0% of total effect). We systematically evaluated the robustness of the mediation effects through adjustments for multiple covariates in separate multivariable Cox regression models. Specifically, we replaced eGFR values with those derived solely from creatinine to assess robustness, as well as analyzed the mediation effect with all-cause mortality as the outcome event.

Multiple confounding factors were adjusted in separate multivariable Cox regression models, with variable selection guided by either statistical significance (*p* < 0.05) in preliminary univariate analyses or established clinical relevance to either prognosis or lesion complexity. Model I was adjusted for age, sex, BMI, hypertension (HTN), diabetes mellitus (DM), smoking, previous PCI, total cholesterol (TC), triglycerides (TG), low density lipoprotein cholesterol (LDL-C), high density lipoprotein cholesterol (HDL-C); Model II was adjusted for age, BMI, DM, heart rate, diuretics, Isu, aspirin, AMI, left ventricular ejection fraction (LVEF); Model III was adjusted for Model I plus Model II. All statistical analyses in the present study were performed using the R Programming Language 4.4.1, and MedCalc 19.1 (MedCalc Software Ltd, Ostend, Belgium). Statistical significance was defined as two-tailed *p* < 0.05.

## 3. Results

### 3.1 Baseline Characteristics

The final analytic cohort meeting the eligibility criteria included 1400 patients (**Supplementary Fig. 1**), with a mean age of 67.1 ± 11.3 years. Baseline characteristics stratified by the incidence of MACEs are presented in Table 1. Participants experiencing MACEs were older and had higher levels of heart rate, cTnT, BNP, serum creatinine, cystatin C, Hcy, as well as higher bSS. They also exhibited lower LVEF, BMI, and eGFR calculated using a combined creatinine-cystatin C method. Compared to patients without MACEs, the MACEs group showed higher rates of AMI, chronic obstructive pulmonary disease (COPD) and DM, increased use of insulin and diuretics, but lower aspirin utilization at discharge. A greater proportion of these patients had eGFR values below 60 mL/min/1.73 m^2^ [85 (37.1%) vs. 282 (24.1%)] or a bSS of 22 or greater [78 (34.1%) vs. 201 (17.2%)], compared to those who did not experience MACEs (all *p*-values were <0.05). Baseline characteristics of the four groups classified by eGFR (<60 vs. ≥60 mL/min/1.73 m^2^) and bSS (<22 vs. ≥22) are detailed in **Supplementary Table 1**.

**Table 1.** **Baseline characteristics**.

Variable		Total population	No MACEs (n = 1171)	MACEs (n = 229)	*p*
Age, years		67.09 ± 11.28	66.31 ± 11.24	71.08 ± 10.63	<0.001
Female, n (%)		405 (28.9)	336 (28.7)	69 (30.1)	0.661
BMI, kg/m^2^		24.39 ± 2.97	24.47 ± 2.96	24.02 ± 2.98	0.036
Smoking, n (%)		742 (53.0)	626 (53.5)	116 (50.7)	0.437
Previous PCI, n (%)		121 (8.6)	94 (8.0)	27 (11.8)	0.064
COPD, n (%)		44 (3.2)	31 (2.7)	13 (5.7)	0.016
Hypertension, n (%)		951 (67.9)	786 (67.1)	165 (72.1)	0.144
DM, n (%)		558 (39.9)	444 (37.9)	114 (49.8)	0.001
Previous stroke, n (%)		66 (4.7)	54 (4.6)	12 (5.2)	0.681
SBP, mmHg		133.14 ± 21.00	133.16 ± 20.91	133.05 ± 21.53	0.942
Heart rate, bpm		7.11 ± 14.32	76.83 ± 14.04	79.60 ± 15.50	0.004
cTnT, pg/mL		40.82 (11.73, 821.15)	34.29 (10.95, 750.80)	101.10 (16.15, 1094.00)	<0.001
BNP, pg/mL		124.65 (43.83, 328.49)	117.90 (40.60, 328.49)	213.40 (74.10, 630.20)	<0.001
Serum creatinine, mg/dL		0.87 (0.74, 1.04)	0.87 (0.74, 1.02)	0.89 (0.74, 1.21)	0.005
Cystatin C, mg/dL		1.14 (0.97, 1.41)	1.13 (0.96, 1.36)	1.27 (1.04, 1.73)	<0.001
eGFR, mL/min/1.73 m^2^		75.62 (58.49, 91.24)	77.13 (61.43, 92.34)	65.02 (43.59, 84.75)	<0.001
eGFR <60 mL/min/1.73 m^2^		367 (25.2)	282 (24.1)	85 (37.1)	<0.001
FBG, mmol/L		6.96 ± 3.21	6.92 ± 3.25	7.17 ± 3.04	0.285
TG, mmol/L		1.52 (1.08, 2.13)	1.55 (1.08, 2.17)	1.44 (1.11, 1.89)	0.203
TC, mmol/L		4.37 (3.62, 5.15)	4.36 (3.64, 5.18)	4.44 (3.55, 5.10)	0.760
HDL-C, mmol/L		1.14 (0.96, 1.30)	1.14 (0.96, 1.30)	1.14 (0.95, 1.31)	0.740
LDL-C, mmol/L		2.67 (2.11, 3.24)	2.67 (2.12, 3.24)	2.68 (2.06, 3.25)	0.829
Hcy, µmol/L		14.15 (11.10, 17.2)	13.90 (11.00, 16.72)	15.60 (11.65, 19.95)	0.001
LVEF		54.99 ± 8.72	55.47 ± 8.32	52.54 ± 10.21	<0.001
AMI, n (%)		702 (50.1)	568 (48.5)	134 (58.5)	0.006
Diagnosis, n (%)					0.001
	UA	698 (49.9)	603 (51.5)	95 (41.5)	
	NSTEMI	304 (21.7)	233 (19.9)	71 (31.0)	
	STEMI	398 (28.4)	335 (28.6)	63 (27.5)	
Aspirin, n (%)		1365 (97.5)	1150 (98.2)	215 (93.9)	<0.001
P_2_Y_12_ receptor inhibitor, n (%)		1379 (98.5)	1154 (98.5)	225 (98.3)	0.737
Statins, n (%)		1372 (98.0)	1148 (98.0)	224 (97.8)	0.828
β-blockers, n (%)		971 (69.4)	815 (69.6)	156 (68.1)	0.658
ACEI/ARB, n (%)		638 (45.6)	530 (45.3)	108 (47.2)	0.597
Diuretics, n (%)		242 (17.3)	172 (14.7)	70 (30.6)	<0.001
Insulin, n (%)		147 (10.5)	107 (9.1)	40 (17.5)	<0.001
bSS ≥22		279 (19.9)	201 (17.2)	78 (34.1)	<0.001
bSS		13.00 (8.00, 20.00)	12.00 (7.00, 19.00)	19.00 (12.00, 26.50)	<0.001

COPD, chronic obstructive pulmonary disease; DM, diabetes mellitus; SBP, systolic blood pressure; FBG, fasting blood glucose; TG, triglyceride; TC, total cholesterol; Hcy, homocysteine; MACEs, major adverse cardiovascular events; PCI, percutaneous coronary intervention; BNP, brain natriuretic peptide; eGFR, estimated glomerular filtration rate; HDL-C, high density lipoprotein cholesterol; LDL-C, low density lipoprotein cholesterol; cTnT, cardiac troponin T; LVEF, left ventricular ejection fraction; NSTEMI, non-ST-elevation myocardial infarction; STEMI, ST-elevation myocardial infarction; AMI, acute myocardial infarction; UA, unstable angina; ACEI/ARB, angiotensin converting enzyme inhibitor/angiotensin receptor blocker; bSS, baseline SYNTAX score; BMI, body mass index.

### 3.2 The Relationship Between Renal Dysfunction and the Complexity of Coronary Artery Lesion

**Fig. 1.**
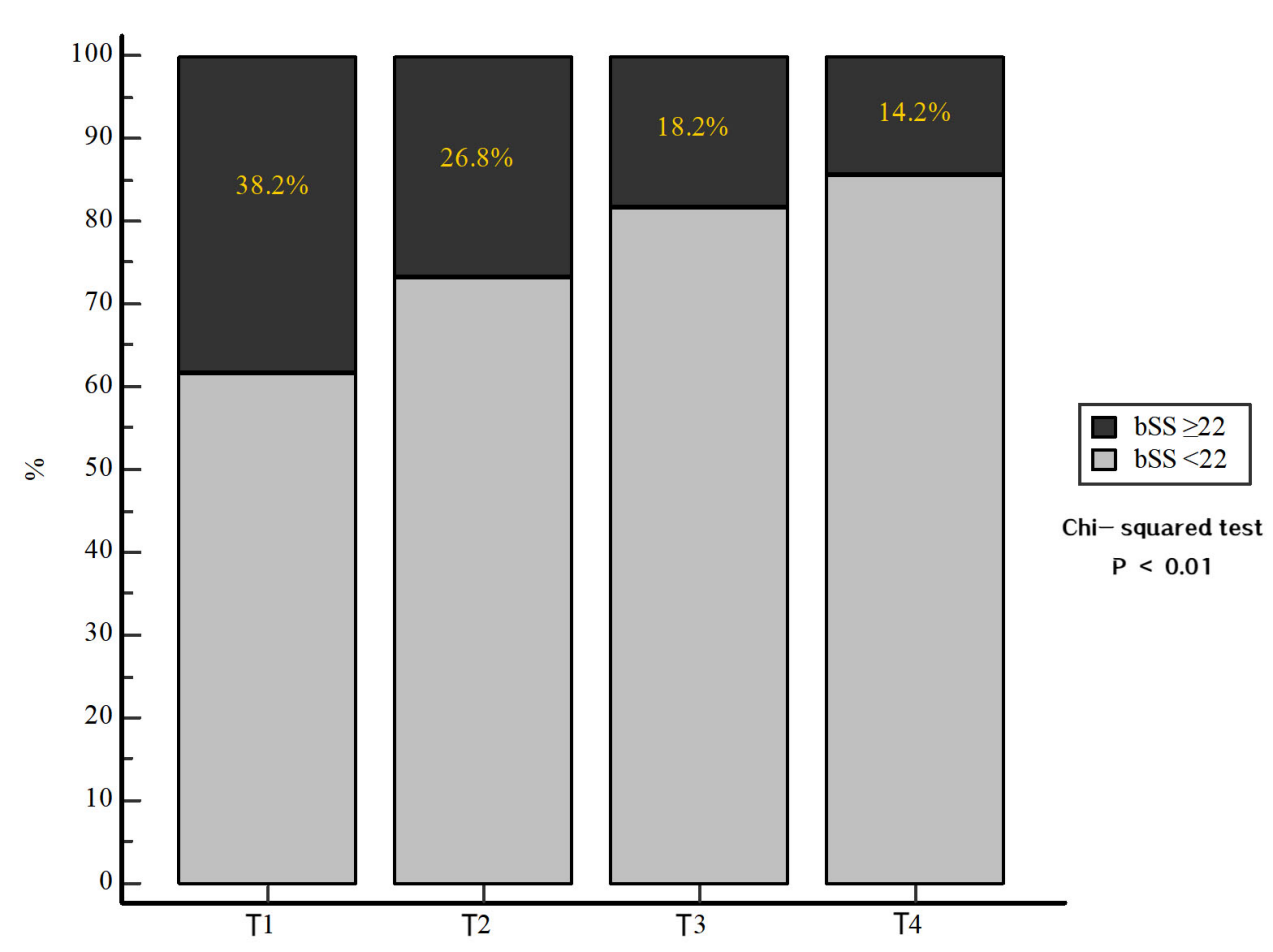
**The proportion of complex lesions (bSS ≥22) in ACS patients based on eGFR groups**. Note: T1: eGFR <30 mL/min/1.73 m^2^; T2: 30 ≤ eGFR < 60 mL/min/1.73 m^2^; T3: 60 ≤ eGFR < 90 mL/min/1.73 m^2^; T4: eGFR ≥90 mL/min/1.73 m^2^. bSS, baseline SYNTAX score; eGFR, estimated glomerular filtration rate; ACS, acute coronary syndrome.

Spearman’s correlation analysis identified a statistically significant negative correlation between eGFR and bSS (r = –0.20, *p* < 0.001, **Supplementary Fig. 2**). Participants were categorized into four groups based on different eGFR levels: T1: eGFR <30 mL/min/1.73 m^2^; T2: 30 ≤ eGFR < 60 mL/min/1.73 m^2^; T3: 60 ≤ eGFR < 90 mL/min/1.73 m^2^; T4: eGFR ≥90 mL/min/1.73 m^2^. Participants with lower eGFR levels (38.2% vs. 26.8%, 18.2% vs. 14.2%, *p* < 0.010, Fig. 1) had a higher proportion of patients with a bSS ≥22.

Multivariate logistic regression analysis revealed that each 10-unit decrease in eGFR was independently associated with an increased likelihood of complex lesions (bSS ≥22), with an odds ratio of 1.121 (95% CI: 1.050–1.197, *p* = 0.001, **Supplementary Table 2**). This association was adjusted for potential confounders, including age, sex, hypertension, BMI, DM, smoking, TC, TG, LDL-C, HDL-C, prior PCI, and homocysteine levels. RCS curve analysis demonstrated a dose-response relationship between eGFR and the risk of complex lesions in ACS patients, after adjusting for the aforementioned variables (*p* for nonlinearity = 0.925, **Supplementary Fig. 3**).

### 3.3 The Relationship Between eGFR, bSS, and Adverse Events

**Fig. 2.**
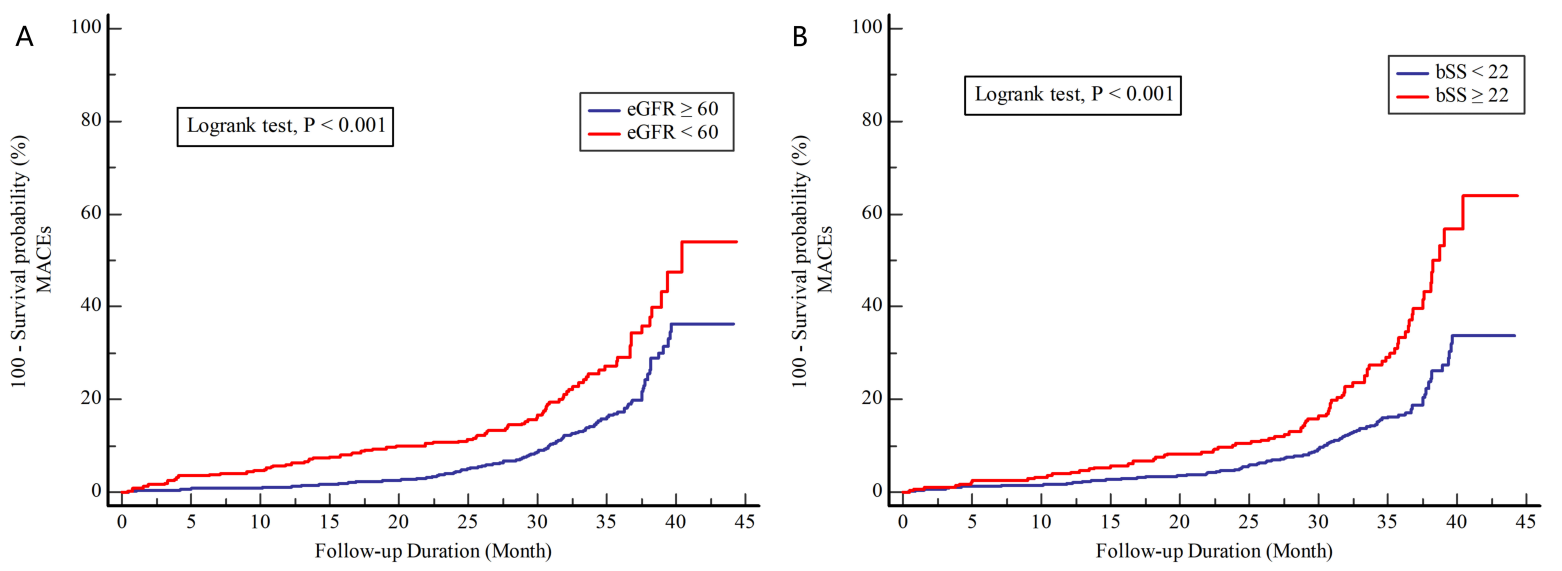
**Cumulative incidence of MACEs stratified by eGFR (A) and bSS (B)**. bSS, baseline SYNTAX score; eGFR, estimated glomerular filtration rate; MACEs, major adverse cardiovascular events.

Over a median follow-up period of 31.03 (27.34, 35.06) months, 229 cases (16.4%) of MACEs were documented, including 99 cases (7.0%) of all-cause death, 41 cases (2.9%) of MI, and 123 cases (8.9%) of unplanned revascularization. The incidence of MACEs, all-cause death, and cardiac death increased with decreased eGFR and elevated bSS (Fig. 2 and **Supplementary Table 3**, **Supplementary Figs. 4,5**). Patients were further stratified into four groups based on eGFR and bSS. Kaplan-Meier curves indicated that individuals with both lower eGFR (<60) and higher bSS (≥22) exhibited the highest incidences of MACEs, all-cause death, and cardiac death (**Supplementary Fig. 6**).

**Fig. 3.**
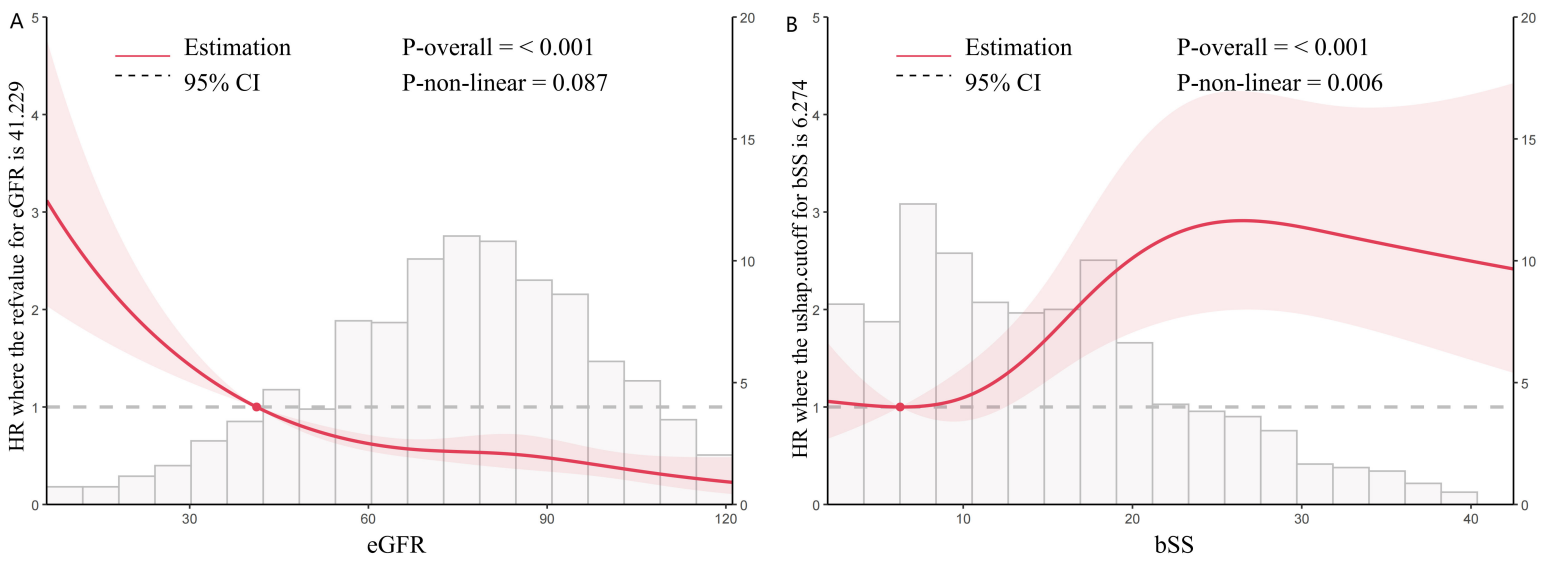
**Dose-responsive relationship of the eGFR (A) and bSS (B) with MACEs**. The RCS curves are derived from a Cox regression adjustment model, which includes factors such as age, sex, BMI, HTN, DM, smoking, previous PCI, TG, TC, LDL-C, HDL-C, HR, diuretics, Isu, aspirin, AMI, LVEF. RCS, restricted cubic spline; bSS, baseline SYNTAX score; eGFR, estimated glomerular filtration rate; MACEs, major adverse cardiovascular events; BMI, body mass index; HTN, hypertension; DM, diabetes mellitus; PCI, percutaneous coronary intervention; TG, triglyceride; TC, total cholesterol; HDL-C, high density lipoprotein cholesterol; LDL-C, low density lipoprotein cholesterol; HR, hazard ratio; AMI, acute myocardial infarction; LVEF, left ventricular ejection fraction.

Univariate Cox regression showed that eGFR, age, BMI, AMI, DM, heart rate, bSS, LVEF, diuretics, and insulin use were risk factors for MACEs, whereas aspirin use was associated with a reduced risk (**Supplementary Table 4**). After adjusting for multiple confounding factors, each 10-unit decrease in eGFR (Model I: HR 1.125, 95% CI: 1.056–1.197, *p* < 0.001; Model II: HR 1.095, 95% CI: 1.029–1.166, *p* = 0.004; Model III: HR 1.093, 95% CI: 1.024–1.166, *p* = 0.007) and 1-unite increase in bSS (Model I: HR 1.037, 95% CI: 1.024–1.050, *p* < 0.001; Model II: HR 1.035, 95% CI: 1.021–1.049, *p* < 0.001; Model III: HR 1.034, 95% CI: 1.020–1.048, *p* < 0.001) were associated with an increased risk of MACEs in ACS patients (Table 2). RCS curve analysis showed eGFR exhibited a negative dose-response relationship with MACEs (non-linear *p* = 0.087), while bSS demonstrated a positive relationship (non-linear *p* = 0.006) (Fig. 3). Similar trends were observed when all-cause death was considered as the outcome (**Supplementary Table 5, Supplementary Fig. 7**).

**Table 2.** **Associations between the eGFR and bSS with MACEs**.

MACEs	eGFR (Per 10-unit decrease)		bSS (Per 1-unit increase)	
	HR (95% CI)	*p*	HR (95% CI)	*p*
Unadjusted Model	1.190 (1.130–1.254)	<0.001	1.042 (1.029–1.054)	<0.001
Adjusted Model I	1.125 (1.056–1.197)	<0.001	1.037 (1.024–1.050)	<0.001
Adjusted Model II	1.095 (1.029–1.166)	0.004	1.035 (1.021–1.049)	<0.001
Adjusted Model III	1.093 (1.024–1.166)	0.007	1.034 (1.020–1.048)	<0.001

Model I was adjusted for age, sex, BMI, HTN, DM, smoking, previous PCI, TG, TC, LDL-C, HDL-C; Model II was adjusted for age, BMI, DM, Heart rate, diuretics, Isu, aspirin, AMI, LVEF; Model III was adjusted for Model I plus Model II. bSS, baseline SYNTAX score; eGFR, estimated glomerular filtration rate; MACEs, major adverse cardiovascular events; BMI, body mass index; HTN, hypertension; DM, diabetes mellitus; PCI, percutaneous coronary intervention; TG, triglyceride; TC, total cholesterol; HDL-C, high density lipoprotein cholesterol; LDL-C, low density lipoprotein cholesterol; HR, hazard ratio; AMI, acute myocardial infarction; LVEF, left ventricular ejection fraction.

### 3.4 Association of eGFR and bSS With Adverse Events in Subgroups

**Fig. 4.**
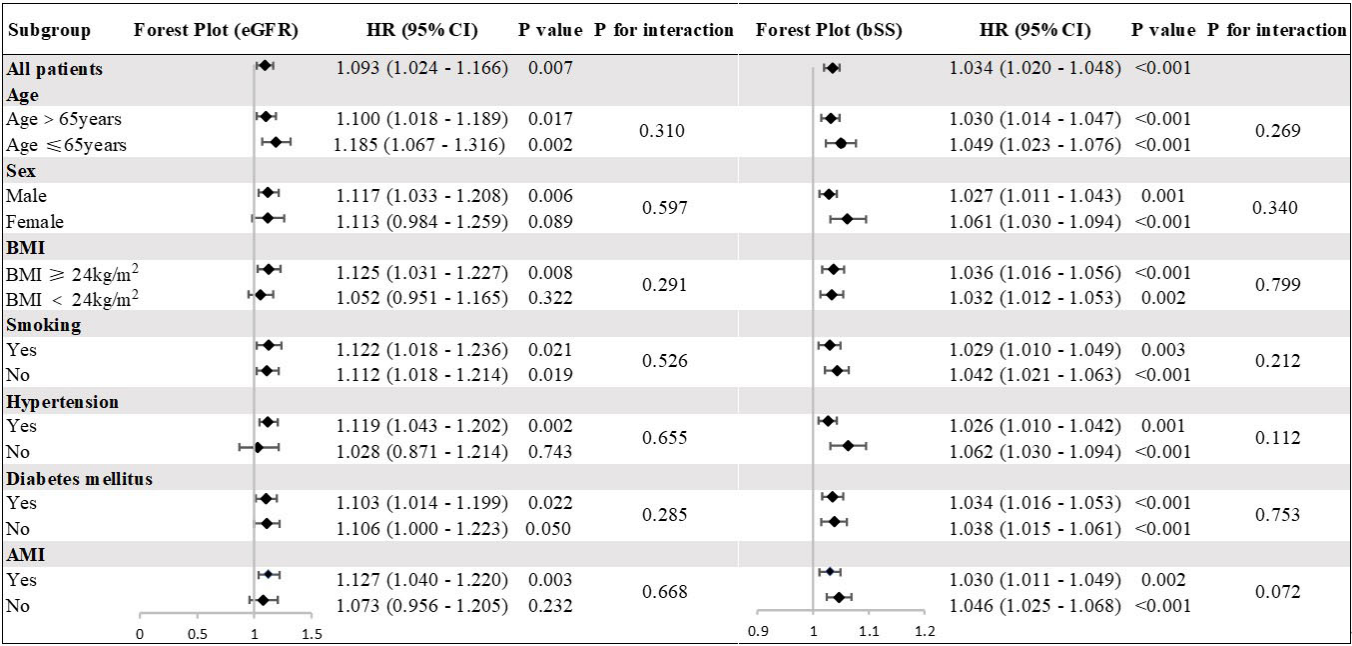
**Subgroup Analyses for MACEs**. The hazard ratio (HR) indicates that each 10-unit decrease in eGFR and each 1-unit increase in bSS correspond to an increased risk. All models were adjusted for age, sex, BMI, HTN, DM, smoking, previous PCI, TG, TC, LDL-C, HDL-C, Heart rate, diuretics, Isu, aspirin, AMI, LVEF. bSS, baseline SYNTAX score; eGFR, estimated glomerular filtration rate; MACEs, major adverse cardiovascular events; BMI, body mass index; HTN, hypertension; DM, diabetes mellitus; PCI, percutaneous coronary intervention; TG, triglyceride; TC, total cholesterol; HDL-C, high density lipoprotein cholesterol; LDL-C, low density lipoprotein cholesterol; AMI, acute myocardial infarction; LVEF, left ventricular ejection fraction.

Subgroup analyses were performed to determine if the predictive power of eGFR and bSS was consistent across different demographic groups and comorbidities (Fig. 4). After adjusting for various confounding variables, we found that both eGFR and bSS were significant indicators of MACEs across various subgroups. In the selected subgroup, no significant association was found with the risk of MACE (all interaction *p*-values were >0.05). Comparable trends were observed when all-cause death was considered as outcome (**Supplementary Fig. 8**).

### 3.5 Mediation Analysis

**Fig. 5.**
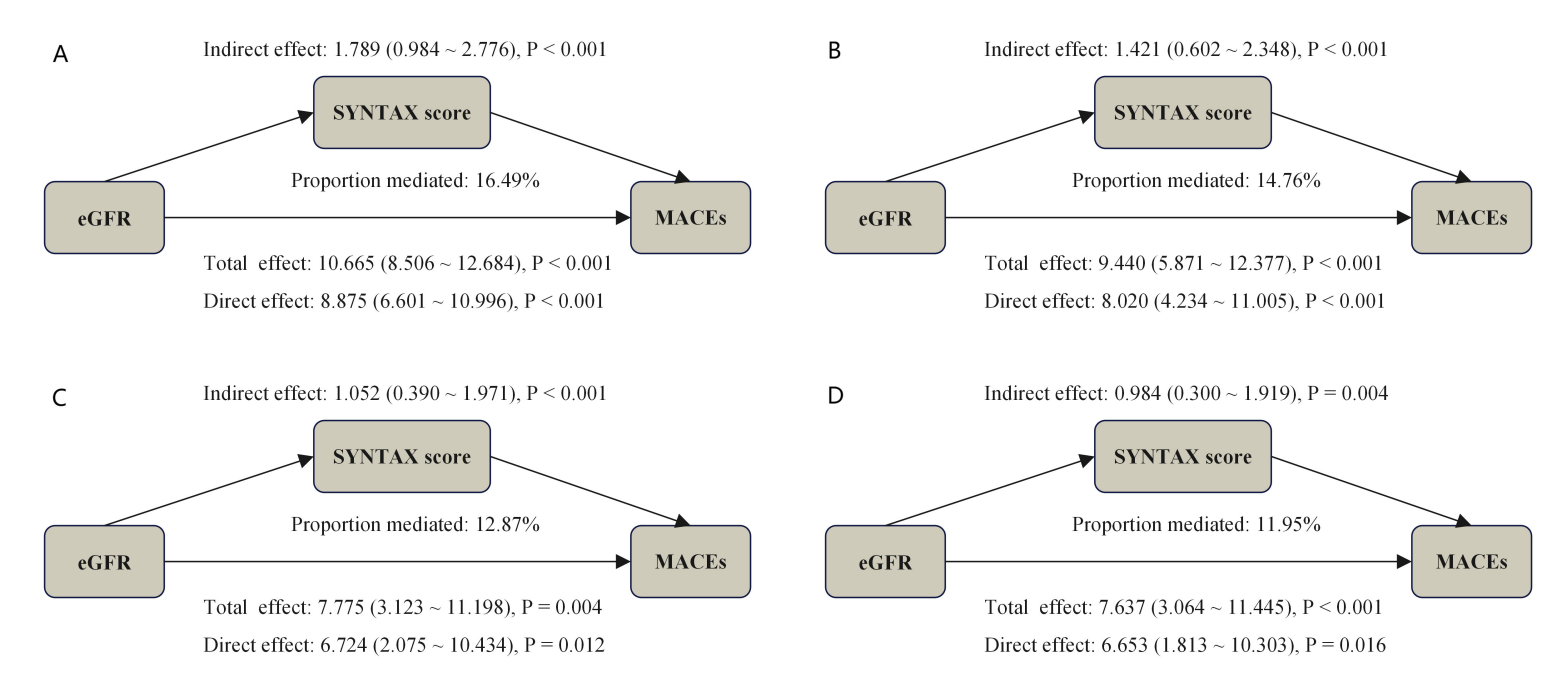
**Causal mediation analysis quantified bSS-mediated pathways in eGFR-MACE associations across covariate-adjusted models**. (A) represents the unadjusted Model; (B) represents the adjusted Model I; (C) represents the adjusted Model II; (D) represents the adjusted Model III. Model I was adjusted for age, sex, BMI, HTN, DM, smoking, previous PCI, TG, TC, LDL-C, HDL-C; Model II was adjusted for age, BMI, DM, Heart rate, diuretics, Isu, aspirin, AMI, LVEF; Model III was adjusted for Model I plus Model II. bSS, baseline SYNTAX score; eGFR, estimated glomerular filtration rate; MACE, major adverse cardiovascular event; BMI, body mass index; HTN, hypertension; DM, diabetes mellitus; PCI, percutaneous coronary intervention; TG, triglyceride; TC, total cholesterol; HDL-C, high density lipoprotein cholesterol; LDL-C, low density lipoprotein cholesterol; AMI, acute myocardial infarction; LVEF, left ventricular ejection fraction.

As illustrated in Fig. 5, the mediation analysis demonstrated that the complexity of coronary artery lesions (bSS) mediated the relationship between renal function (represented by eGFR) and the occurrence of MACEs across various adjusted models. Specifically, bSS accounted for mediation proportions of 16.49%, 14.76%, 12.87%, and 11.95% in unadjusted Model, adjusted Model I, adjusted Model II, and adjusted Model III analyses, respectively. Furthermore, we tested the robustness of our findings by substituting eGFR values derived solely from creatinine. The mediation proportions were 20.71%, 20.30%, 17.20%, and 15.66% across the respective analyses (**Supplementary Fig. 9**). Similar mediating effects were noted when endpoint was defined as all-cause death (**Supplementary Fig. 10**).

## 4. Discussion

In this retrospective cohort study of 1400 patients with ACS followed for up to 31.03 months, a lower eGFR (calculated using the 2021 CKD-EPI equation with creatinine and cystatin C) and a higher bSS were significantly associated with an increased risk of MACEs after PCI. These associations remained significant after adjusting for established cardiovascular risk factors across models and subgroups. Moreover, the study suggested that an elevated bSS partially mediated the relationship between renal dysfunction and adverse cardiovascular outcomes in ACS patients.

Previous research has demonstrated that an extended duration of CKD, particularly in patients progressing to dialysis, is associated with an increased likelihood of complex coronary artery lesions (bSS ≥22) and significant calcification [16, 17, 18, 19, 20, 21, 22, 27]. Moreover, even after adjusting for known risk factors such as diabetes and hypertension, the risk of mortality progressively increases with the worsening of CKD. Patients in CKD stages G3a to G4 (15–60 mL/min/1.73 m^2^) have approximately double and triple the cardiovascular disease (CVD) mortality risk, respectively, compared to those without CKD [2, 28]. Our study revealed a significant dose-response relationship between lower eGFR and complex coronary lesions in ACS patients, persisting after adjustment (p for nonlinearity = 0.925). Each 10 mL/min/1.73 m^2^ reduction in eGFR was independently associated with a higher risk of complex lesions (OR: 1.121, 95% CI: 1.050–1.197, *p* = 0.001) and long-term adverse cardiovascular events, aligning with prior evidence. Our study employed the 2021 CKD-EPI equation, which integrates both serum creatinine and cystatin C to calculate eGFR. This method offers a more precise assessment of kidney function [25, 26] and may better reveal the relationship between renal function and cardiovascular outcomes compared to the traditional equation based solely on creatinine.

Furthermore, complex coronary artery lesions are linked to increased risks of perioperative complications, target lesion failure, recurrent MI, and in-stent restenosis post-PCI [29, 30], all of which collectively increase the incidence of adverse events. Our study revealed a significant positive dose-response relationship between elevated bSS and adverse cardiovascular events. Notably, a non-linear association was observed between bSS and MACE, whereas all-cause mortality exhibited a linear dose-response pattern. This discrepancy may be attributed to the composition of the composite MACE endpoint, in which unplanned revascularization accounted for 53.71% (123/229) of events. Theoretically, while increasing lesion complexity increases the likelihood of requiring late revascularization, patients with highly complex lesions may face technical barriers to revascularization and consequently opt for conservative management, potentially explaining the non-linear bSS-MACE relationship.

This study provides the first evidence that coronary lesion complexity partially mediates the association between renal dysfunction and adverse cardiovascular outcomes, shedding light on the pathophysiological interplay between CKD and coronary atherosclerosis. This mediation may be attributed to several factors. On one hand, CKD exacerbates calcium-phosphate metabolism disorders and inflammatory responses. These, along with associated metabolic risk factors such as obesity, diabetes, hypertension, and lipid metabolism disorders, collectively promote the progression of coronary atherosclerosis and vascular calcification [5, 11, 12, 13]. In addition, non-traditional risk factors also play a potential role in advancing atherosclerosis [5, 31]. For example, studies have revealed that enhanced tyrosine sulfation [31], as well as p-cresyl sulfate (PCS) [32], is associated with CKD-related atherosclerosis. Furthermore, a prospective cohort study (the CRUISE-MET study, NCT06383208) utilized lipidomics to investigate non-traditional lipid metabolites that promote the progression of coronary lesions in CKD patients [33]. Nevertheless, the mechanisms responsible for CKD-specific atherosclerosis are still not completely understood and warrant further study.

In summary, routine assessment of renal function using the 2021 CKD-EPI equation (incorporating creatinine and cystatin C) [25, 26] is critical for ASCVD risk management, and allows early identification of high-risk CKD populations. In high-risk populations, targeted use of cardiorenal protective agents—including finerenone [34], glucagon-like peptide-1 (GLP-1) receptor agonists [35], and sodium-glucose cotransporter-2 (SGLT-2) inhibitors [36]—may attenuate the progression of atherosclerosis [14, 37]. Furthermore, current ACS risk stratification tools (e.g., GRACE score) rely solely on creatinine-based renal estimates and omit coronary lesion complexity, limiting their utility in personalized care [38, 39, 40]. To address this gap, development of AI-enhanced tools integrating eGFR, bSS, and novel biomarkers [41] could better predict post-PCI MACE [39, 41]. Specifically for renal-impaired patients, combining renal function and coronary lesion assessments—guided by intravascular imaging or functional testing—may optimize revascularization strategies and clinical outcomes.

### Limitations 

While this study provides valuable insights, it also presents certain limitations that warrant consideration. First, the single-center observational design limits causal inference between eGFR, bSS, and cardiovascular outcomes, and residual confounding may persist despite adjustment for known risk factors. Nevertheless, this limitation has been partially mitigated through subgroup analyses and sensitivity analyses. Secondly, although the 2021 CKD-EPI equation (combining creatinine and cystatin C) improved the accuracy for estimating GFR, reliance on a single in-hospital eGFR measurement may introduce bias due to fluctuations in short-term renal function. Future studies should incorporate repeated eGFR measurements during follow-up or use urinary biomarkers (e.g., albumin-to-creatinine ratio) to better capture trends in renal function [42]. Finally, since we exclusively used a Chinese cohort, validation across diverse ethnic populations is needed to confirm generalizability.

## 5. Conclusion

This study demonstrates that a lower eGFR and higher bSS are significant predictors of MACEs after PCI in patients with ACS. It also suggests that coronary lesion complexity partially mediate the relationship between renal dysfunction and adverse cardiovascular outcomes in ACS patients. Clinically, these findings underscore the importance of integrating assessments of kidney function with coronary anatomical evaluations to develop individualized treatment strategies for high-risk patients.

## Availability of Data and Materials

The datasets used and analyzed in the study are available from the corresponding author upon reasonable request.

## Supplementary Material

Supplementary material associated with this article can be found, in the online version, at https://doi.org/10.31083/RCM38389.
