# Supplementary material for: Renal Dysfunction, Coronary Artery Lesion Complexity, and Adverse Cardiovascular Outcomes in Patients With Acute Coronary Syndrome
Source: Rev Cardiovasc Med. 2025 Jul 23;26(7):38389. doi: 10.31083/RCM38389 (PMC12326429; doi:10.31083/RCM38389)
Supplement: Supplementary file 1 [file 2153-8174-26-7-38389-s1.docx]

Supplementary Table 1. Baseline characteristics classified by eGFR (<60 vs. ≥60 mL/min/1.73m²) and bSS (<22 vs. ≥22)

| Variable | eGFR ≥ 60 | | eGFR < 60 | | P |
| --- | --- | --- | --- | --- | --- |
|  | bSS < 22  (n = 860) | bSS ≥ 22  (n = 173) | bSS < 22  (n = 261) | bSS ≥ 22  (n = 106) |  |
| Age, years | 64.24 ± 11.03 | 66.96 ± 10.28 | 73.52 ± 8.98 | 74.59 ± 10.02 | <0.001 |
| Female, n (%) | 213 (24.77) | 45 (26.01) | 108 (41.38) | 39 (36.79) | <0.001 |
| BMI, kg/m^2^ | 24.46 ± 2.94 | 24.14 ± 2.97 | 24.58 ± 2.98 | 23.81 ± 3.17 | 0.077 |
| Smoking, n (%) | 476 (55.35) | 103 (59.54) | 120 (45.98) | 43 (40.57) | <0.001 |
| Previous PCI, n (%) | 68 (7.91) | 10 (5.78) | 33 (12.64) | 10 (9.43) | 0.051 |
| COPD, n (%) | 19 (2.21) | 3 (1.73) | 15 (5.75) | 7 (6.60) | 0.004 |
| Hypertension, n (%) | 533 (61.98) | 117 (67.63) | 214 (81.99) | 87 (82.08) | <0.001 |
| DM | 294 (34.19) | 76 (43.93) | 130 (49.81) | 58 (54.72) | <0.001 |
| Previous Stroke, n (%) | 32 (3.72) | 4 (2.31) | 19 (7.28) | 11 (10.38) | 0.001 |
| SBP, mmHg | 132.90 ± 19.84 | 132.90 ± 21.75 | 134.54 ± 23.02 | 132.06 ± 23.76 | 0.664 |
| HR, bpm | 76.32 ± 13.08 | 79.38 ± 15.53 | 77.35 ± 16.92 | 79.27 ± 14.49 | 0.023 |
| cTnT, pg/ml | 37.50 (9.78,1211.90) | 76.70 (14.00,1256.00) | 52.10 (17.40,652.60) | 505.50 (40.25,1867.25) | <0.001 |
| BNP, pg/ml | 94.35 (32.80,288.90) | 138.00 (46.80,328.49) | 204.30 (74.60,601.80) | 350.70 (157.67,1075.12) | <0.001 |
| Serum creatinine, mg/dL | 0.81 (0.70,0.92) | 0.81 (0.72,0.91) | 1.17 (1.02,1.43) | 1.24 (1.05,1.63) | <0.001 |
| Cystatin C, mg/dL | 1.05 (0.93,1.18) | 1.09 (0.96,1.22) | 1.65 (1.48,2.00) | 1.79 (1.51,2.27) | <0.001 |
| eGFR, mL/min/1.73m^2^ | 84.02 (73.49,96.12) | 80.33 (71.80,92.61) | 46.75 (35.79,54.98) | 43.80 (31.38,53.84) | <0.001 |
| FBG, mmol/L | 6.70 ± 2.82 | 7.13 ± 3.26 | 7.37 ± 3.72 | 7.81 ± 4.38 | <0.001 |
| TG, mmol/L | 1.52 (1.09,2.15) | 1.60 (1.08,2.10) | 1.55 (1.12,2.21) | 1.40 (1.02,1.88) | 0.445 |
| TC, mmol/L | 4.34 (3.62,5.17) | 4.54 (3.94,5.80) | 4.26 (3.52,4.84) | 4.29 (3.30,5.02) | <0.001 |
| HDL-C, mmol/L | 1.14 (0.97,1.30) | 1.16 (1.00,1.35) | 1.13 (0.93,1.29) | 1.10 (0.86,1.29) | 0.042 |
| LDL-C, mmol/L | 2.64 (2.11,3.27) | 2.83 (2.32,3.66) | 2.63 (2.05,2.97) | 2.58 (1.81,3.19) | <0.001 |
| Hcy, µmol/L | 12.90 (10.50,16.70) | 13.30 (10.60,16.72) | 16.72 (14.40,21.60) | 18.15 (14.55,22.65) | <0.001 |
| LVEF | 56.23 ± 7.70 | 53.35 ± 8.89 | 54.34 ± 9.56 | 49.23 ± 10.97 | <0.001 |
| AMI, n (%) | 402 (46.74) | 99 (57.23) | 129 (49.43) | 72 (67.92) | <0.001 |
| Diagnosis, n (%) |  |  |  |  | <0.001 |
| UA | 458 (53.26) | 74 (42.77) | 132 (50.57) | 34 (32.08) |  |
| NSTEMI | 151 (17.56) | 44 (25.43) | 72 (27.59) | 37 (34.91) |  |
| STEMI | 251 (29.19) | 55 (31.79) | 57 (21.84) | 35 (33.02) |  |
| Aspirin, n (%) | 849 (98.72) | 172 (99.42) | 256 (98.08) | 104 (98.11) | 0.614 |
| P_2_Y_12_ receptor inhibitor, n (%) | 846 (98.37) | 172 (99.42) | 257 (98.47) | 104 (98.11) | 0.791 |
| Statins, n (%) | 846 (98.37) | 170 (98.27) | 253 (96.93) | 103 (97.17) | 0.386 |
| β-blockers, n (%) | 596 (69.30) | 125 (72.25) | 175 (67.05) | 75 (70.75) | 0.697 |
| ACEI/ARB, n (%) | 380 (44.19) | 75 (43.35) | 132 (50.57) | 51 (48.11) | 0.270 |
| Diuretics, n (%) | 87 (10.12) | 33 (19.08) | 80 (30.65) | 42 (39.62) | <0.001 |
| Insulin, n (%) | 64 (7.44) | 17 (9.83) | 47 (18.01) | 19 (17.92) | <0.001 |
| bSS | 11.00 (7.00,16.00) | 27.50 (24.50,31.30) | 12.00 (8.00,16.50) | 28.00 (24.62,32.00) | <0.001 |

**Abbreviations:** COPD, chronic obstructive pulmonary disease; DM, diabetes mellitus; HR, heart rate; FBG, fasting blood glucose; TG, triglyceride; TC, total cholesterol; Hcy, homocysteine.

Supplementary Table 2. Multivariate logistic regression analysis for predicting complex lesions (bSS≥22)

| Variables | Multivariate logistic analysis | | |
| --- | --- | --- | --- |
|  | OR | 95%CI | P |
| Age | 1.017 | 1.002 - 1.033 | 0.030 |
| Female | 0.906 | 0.630 - 1.303 | 0.595 |
| BMI | 0.939 | 0.894 - 0.987 | 0.013 |
| Smoking | 1.153 | 0.829 - 1.602 | 0.399 |
| Previous PCI | 0.778 | 0.464 - 1.304 | 0.341 |
| Hypertension | 1.131 | 0.820 - 1.559 | 0.453 |
| DM | 1.447 | 1.048 - 1.999 | 0.025 |
| eGFR | 1.121 | 1.050 - 1.197 | 0.001 |
| FBG | 1.022 | 0.977 - 1.070 | 0.347 |
| TC | 1.851 | 0.983 - 3.485 | 0.057 |
| TG | 0.908 | 0.785 - 1.051 | 0.196 |
| HDL-C | 0.478 | 0.219 - 1.045 | 0.065 |
| LDL-C | 0.651 | 0.301 - 1.407 | 0.275 |
| Hcy | 1.006 | 0.998 - 1.014 | 0.122 |

**Abbreviations:** DM, diabetes mellitus; FBG, fasting blood glucose; TG, triglyceride; TC, total cholesterol; odds ratios (OR) indicate an increased risk for each 10-unit decrease in eGFR.

Supplementary Table 3. Comparison of Long-Term Adverse Prognosis Stratified by eGFR and bSS Categories

| Variable | eGFR | | P | bSS | | P |
| --- | --- | --- | --- | --- | --- | --- |
|  | eGFR≥60 (n=1033) | eGFR ＜60 (n=367) |  | bSS＜ 22  (n=1121) | bSS≧22  (n=279) |  |
| MACEs, n (%) | 144 (13.9) | 85 (23.2) | ＜0.001 | 151 (13.5) | 78 (28.0) | ＜0.001 |
| Death or MI, n (%) | 73 (7.1) | 62 (16.9) | ＜0.001 | 88 (7.9) | 47 (16.8) | ＜0.001 |
| All-cause death, n (%) | 49 (4.7) | 50 (13.6) | ＜0.001 | 65 (5.8) | 34 (12.2) | ＜0.001 |
| Cardiac death, n (%) | 27 (2.6) | 33 (9.0) | ＜0.001 | 41 (3.7) | 19 (6.8) | 0.020 |
| MI, n (%) | 29 (2.8) | 12 (3.3) | 0.652 | 26 (2.3) | 15 (5.4) | 0.007 |
| Unplanned revascularization, n (%) | 89 (8.6) | 34 (9.3) | 0.706 | 81 (7.2) | 42 (15.1) | ＜0.001 |
| Stroke, n (%) | 35 (3.4) | 14 (3.8) | 0.703 | 40 (3.6) | 9 (3.2) | 0.781 |

**Supplementary Table 4. Univariate Cox regression analysis for MACEs.**

| Variables | Univariate analysis | | |
| --- | --- | --- | --- |
|  | HR | 95%CI | P |
| Age | 1.042 | 1.028 - 1.055 | <0.001 |
| Female | 1.067 | 0.808 - 1.415 | 0.652 |
| BMI | 0.939 | 0.897 - 0.982 | 0.006 |
| AMI | 1.456 | 1.119 - 1.894 | 0.005 |
| Smoking | 0.918 | 0.708 - 1.189 | 0.516 |
| Previous PCI | 1.350 | 0.903 - 2.018 | 0.143 |
| Hypertension | 1.244 | 0.932 - 1.660 | 0.139 |
| DM | 1.508 | 1.163 - 1.954 | 0.002 |
| SBP | 0.999 | 0.992 - 1.005 | 0.657 |
| Heart rate | 1.013 | 1.005 - 1.022 | 0.003 |
| eGFR | 1.234 | 1.172 - 1.298 | <0.001 |
| FBG | 1.023 | 0.985 - 1.062 | 0.241 |
| TC | 0.983 | 0.886 - 1.091 | 0.751 |
| TG | 0.893 | 0.794 - 1.004 | 0.059 |
| HDL-C | 0.911 | 0.585 - 1.420 | 0.681 |
| LDL-C | 0.978 | 0.848 - 1.128 | 0.761 |
| Hcy | 1.001 | 0.996 - 1.007 | 0.604 |
| bSS | 1.052 | 1.039 - 1.065 | <0.001 |
| LVEF | 0.970 | 0.958 - 0.983 | <0.001 |
| aspirin | 0.325 | 0.144 - 0.732 | 0.007 |
| P2Y12 receptor inhibitor | 0.781 | 0.291 - 2.101 | 0.625 |
| β-blockers | 0.946 | 0.714 - 1.252 | 0.699 |
| Diuretics | 2.350 | 1.774 - 3.113 | <0.001 |
| ACEI/ARB | 1.060 | 0.818 - 1.375 | 0.660 |
| Insulin | 1.841 | 1.309 - 2.590 | 0.001 |

**Abbreviations:** DM, diabetes mellitus; SBP, systolic blood pressure; FBG, fasting blood glucose; TG, triglyceride; TC, total cholesterol. Hazard ratio (HR) indicates increased risk per 10-unit decrease in eGFR and per 1-unit increase in bSS.

**Supplementary Table 5. Associations between the eGFR and bSS with all-cause death.**

| Death | eGFR (Per 10-unit decrease) | |  | bSS (Per 1-unit increase) | |
| --- | --- | --- | --- | --- | --- |
|  | HR (95%CI) | P |  | HR (95%CI) | P |
| Unadjusted Model | 1.350 (1.251 - 1.458) | <0.001 |  | 1.035 (1.016 - 1.054) | <0.001 |
| Adjusted Model I | 1.261 (1.149 - 1.385) | <0.001 |  | 1.029 (1.009 - 1.050) | 0.004 |
| Adjusted Model II | 1.191 (1.083 ~ 1.311) | <0.001 |  | 1.024 (1.003 ~ 1.046) | 0.023 |
| Adjusted Model III | 1.202 (1.087 ~ 1.329) | <0.001 |  | 1.024 (1.002 ~ 1.046) | 0.029 |

Model I was adjusted for age, sex, BMI, HTN, DM, smoking, previous PCI, TG, TC, LDL-C, HDL-C; Model II was adjusted for age, BMI, DM, Heart rate, Diuretics, Isu, aspirin, AMI, LVEF; Model III was adjusted for Model I plus Model II.

**Supplementary Fig. 1. Flowchart of the patient registration**


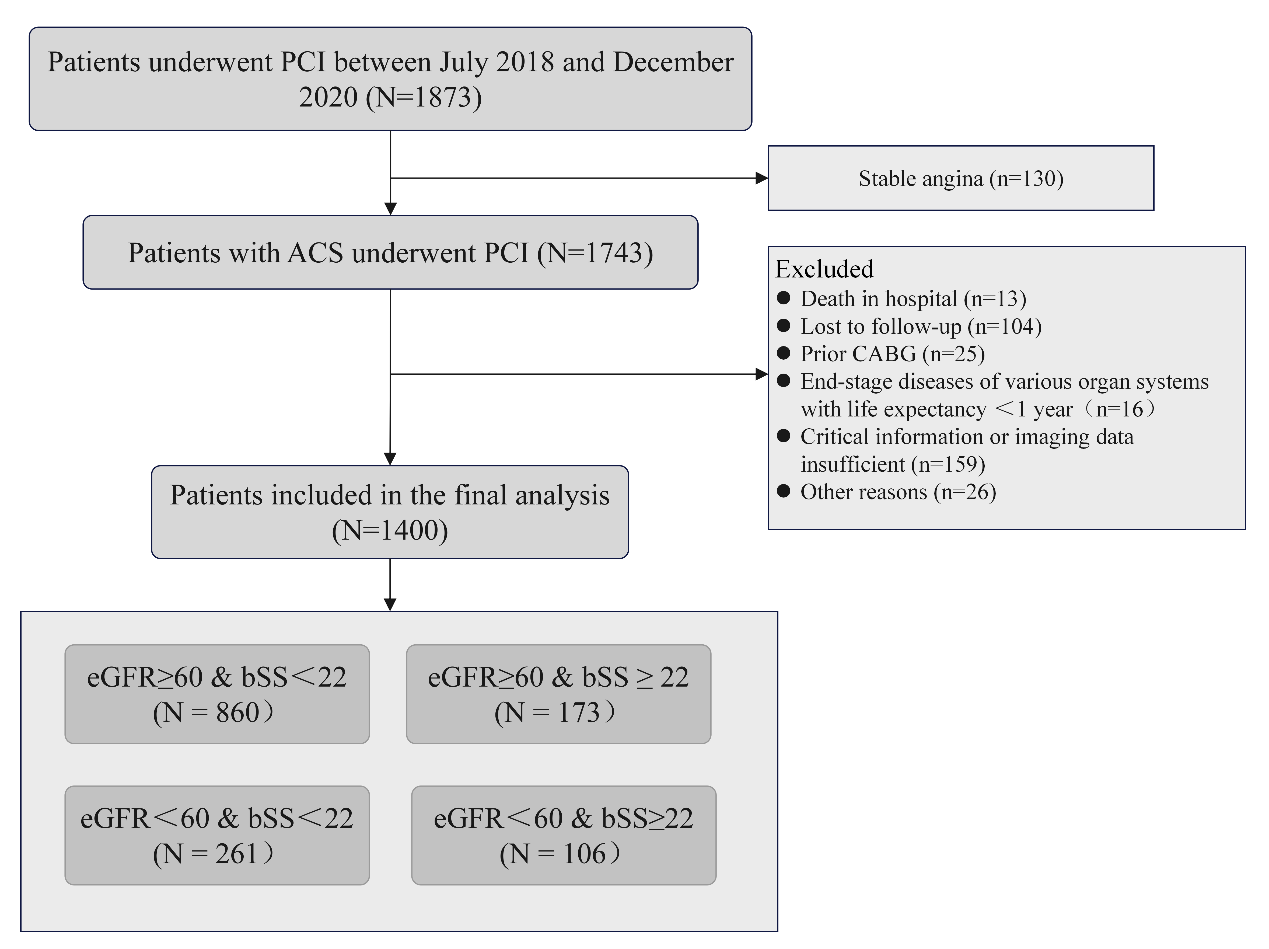


Supplementary Fig. 2. Correlation of bSS with the eGFR. Spearman’s correlation analysis found that there was a negative correlation between the eGFR and bSS (r=-0.20, P<0.001).


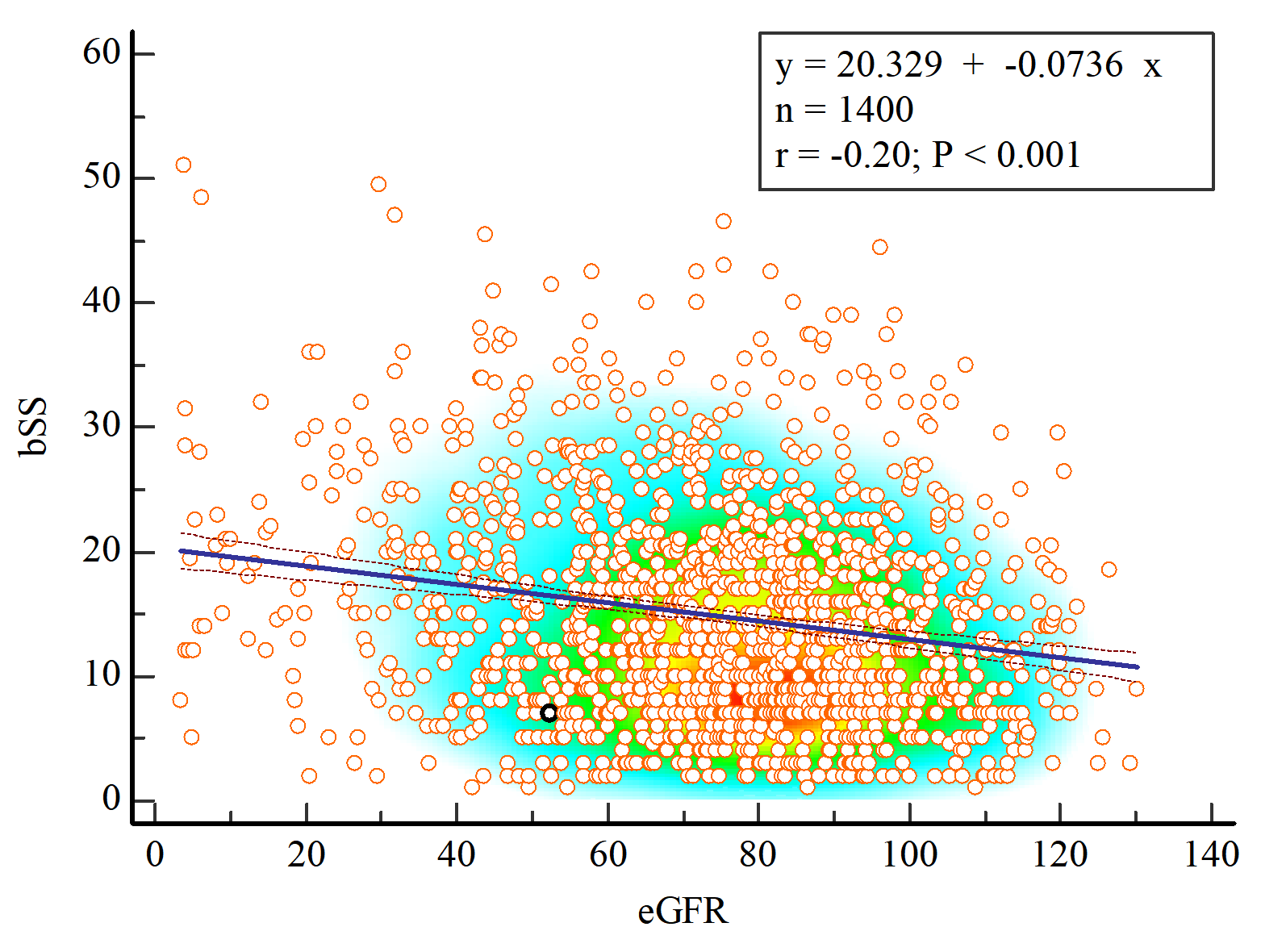


**Supplementary Fig. 3. Dose-responsive relationship of the eGFR and complex lesion in ACS undergoing PCI.** The figure illustrates the restricted cubic spline (RCS) curve derived from a logistic regression adjustment model, highlighting the association between eGFR and complex lesion (bSS ≥ 22). The model adjusts for variables including age, sex, hypertension, BMI, diabetes mellitus, smoking status, TC, TG, LDL-C, HDL-C, previous PCI, and homocysteine.


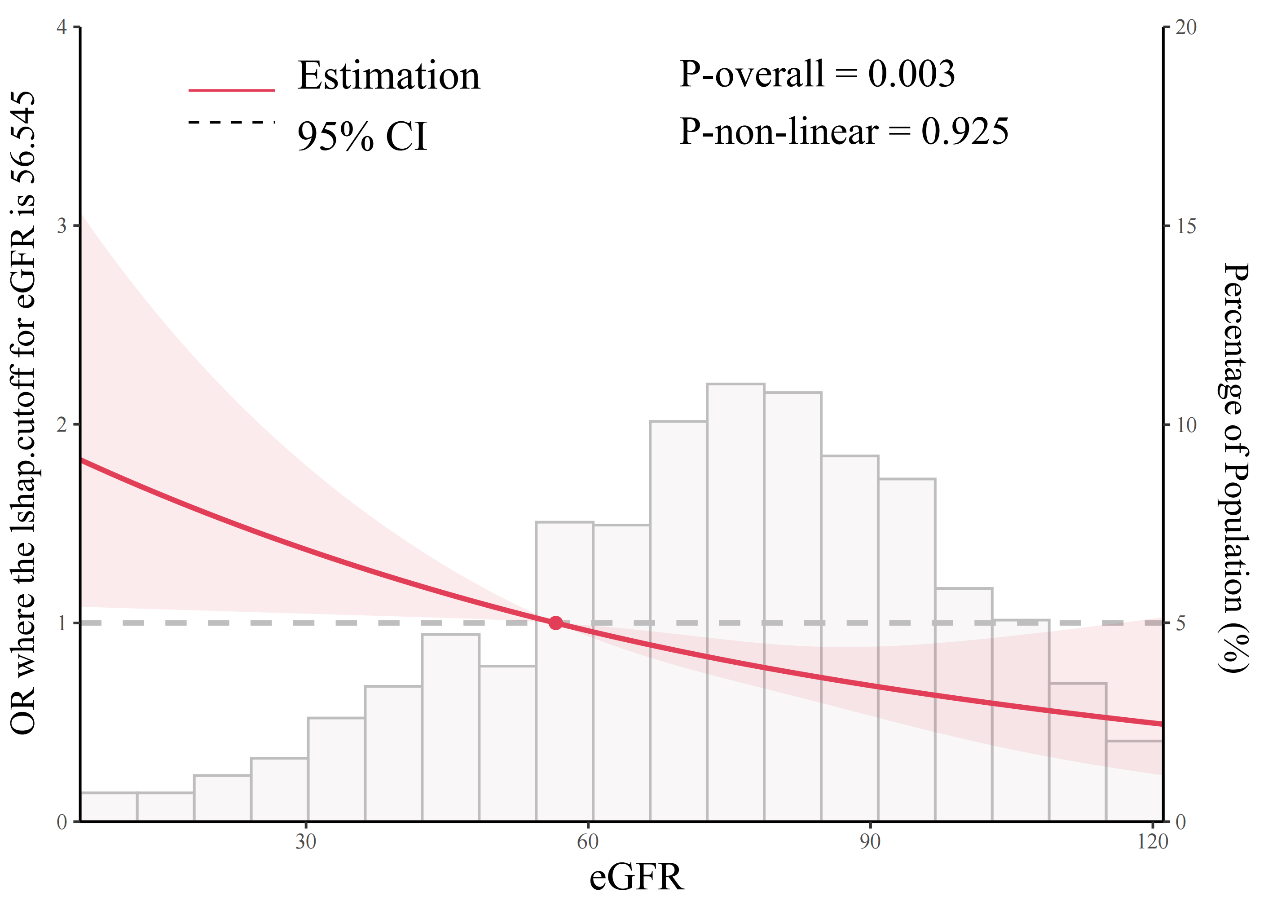


**Supplementary Fig. 4. Cumulative incidence of various secondary endpoints during follow-up, stratified by eGFR.** It includes four endpoint events: all-cause death (A), cardiac death (B), and MI (C), and unplanned revascularization (D).


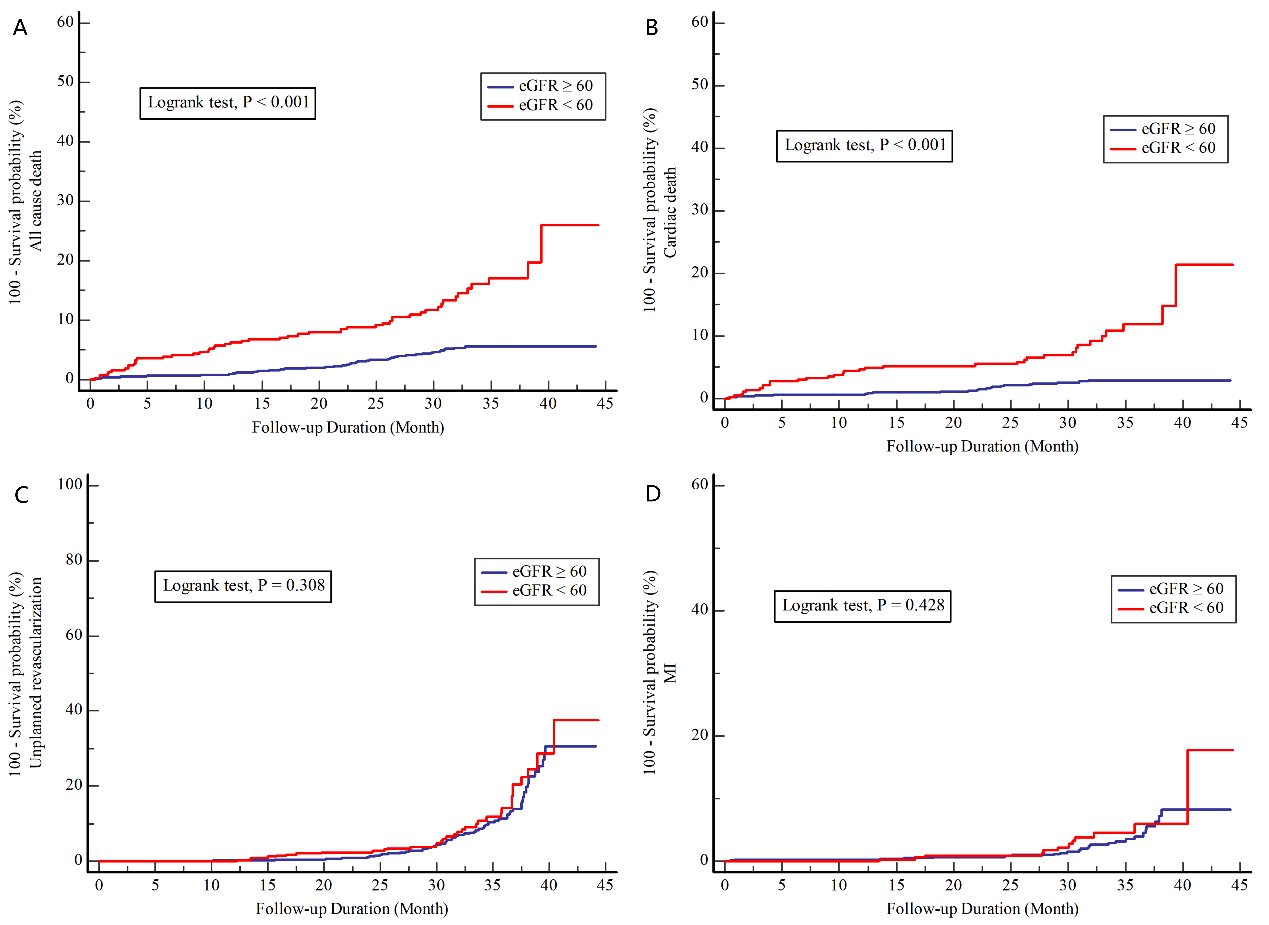


**Supplementary Fig. 5. Cumulative incidence of various secondary endpoints during follow-up, stratified by bSS.** It includes four endpoint events: all-cause death (A), cardiac death (B), and MI (C), and unplanned revascularization (D).


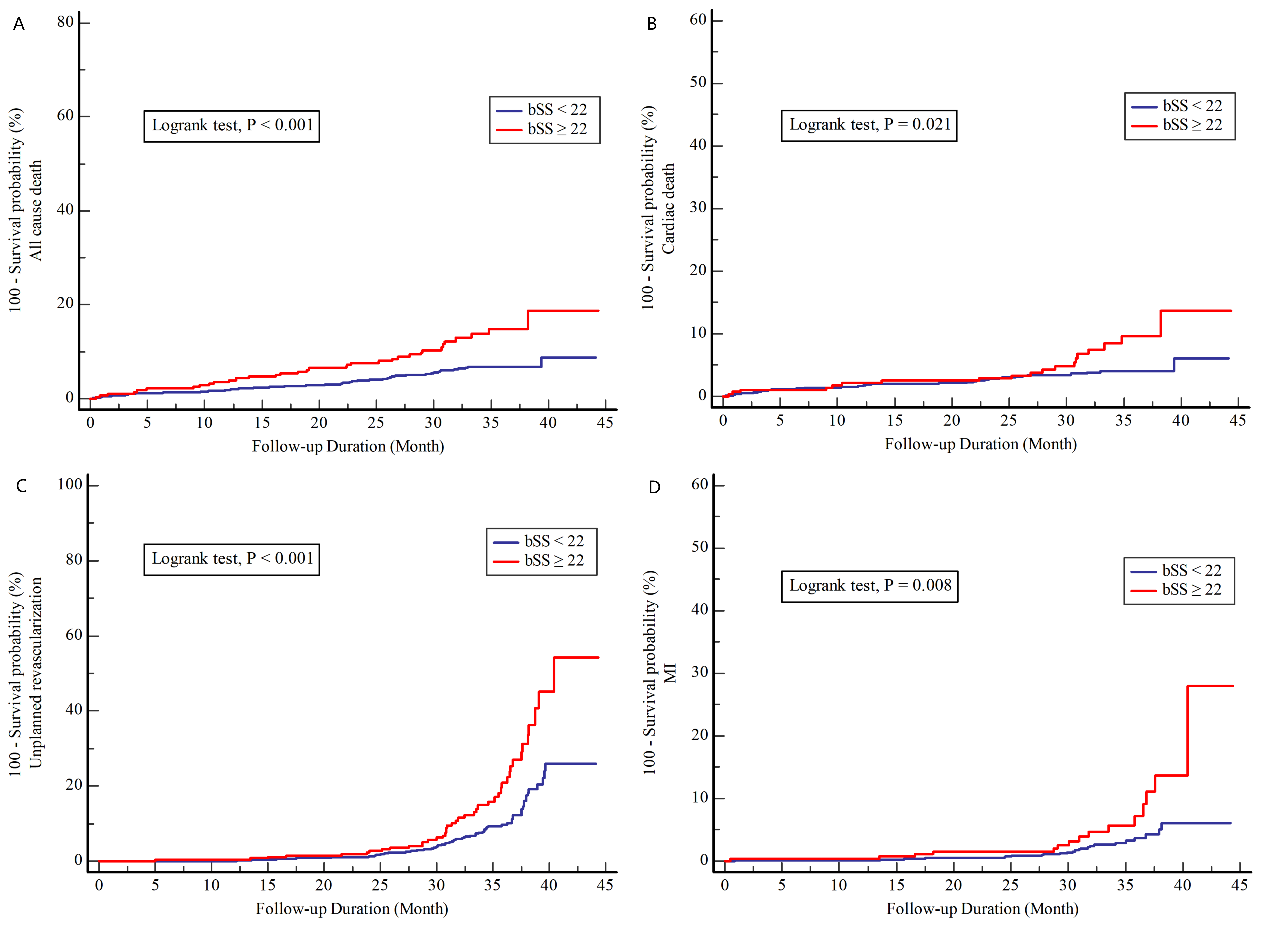


**Supplementary Fig. 6. Cumulative incidence of endpoints during follow-up stratified by bSS and eGFR.** It includes six endpoint events: MACEs (A), all-cause death (B), cardiac death (C), and MI (D), unplanned revascularization (E), and stroke (F).


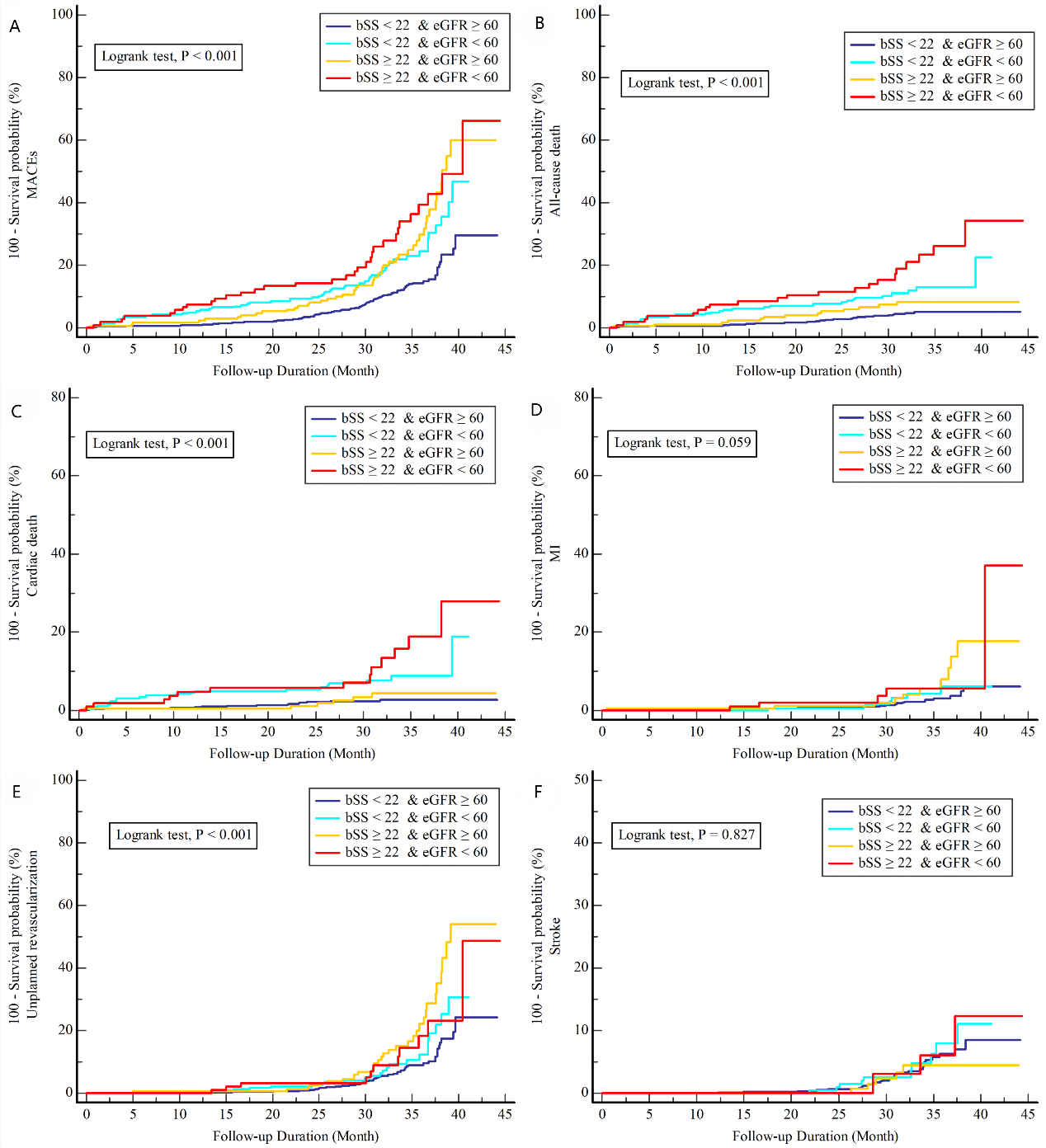


**Supplementary Fig. 7. Dose-responsive relationship of the eGFR(A) and bSS (B) with the risk of with Death in ACS undergoing PCI.**

The restricted cubic spline (RCS) curves are derived from a Cox regression adjustment model, which includes factors such as age, sex, BMI, HTN, DM, smoking, previous PCI, TG, TC, LDL-C, HDL-C, heart rate, Diuretics, Isu, aspirin, AMI, LVEF.


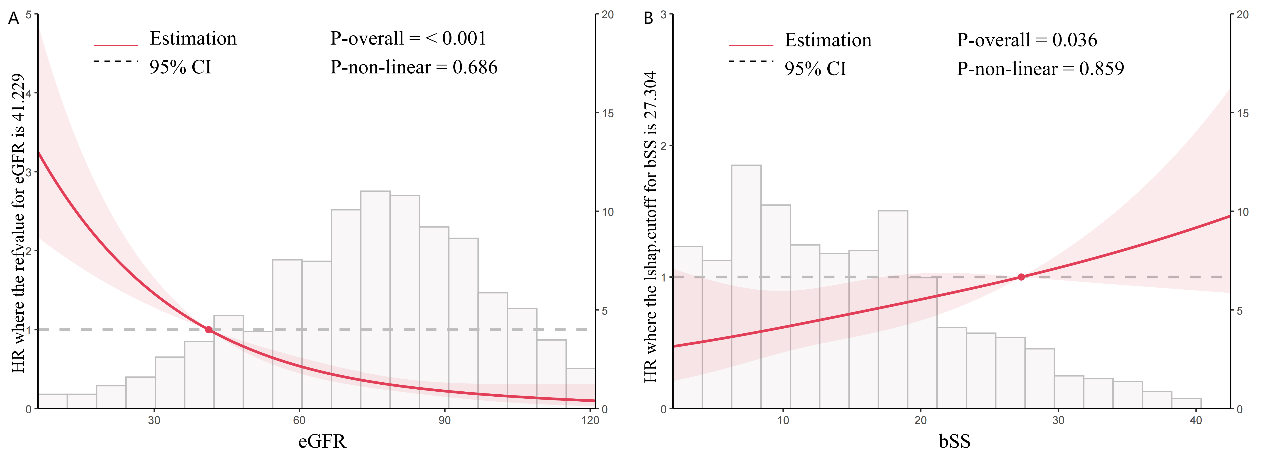


**Supplementary Fig. 8. Subgroup Analyses for death**

Hazard ratio (HR) indicates an increased risk for each 10-unit decrease in eGFR. All models were adjusted for age, sex, BMI, HTN, DM, smoking, previous PCI, TG, TC, LDL-C, HDL-C, heart rate, Diuretics, Isu, aspirin, AMI, LVEF.


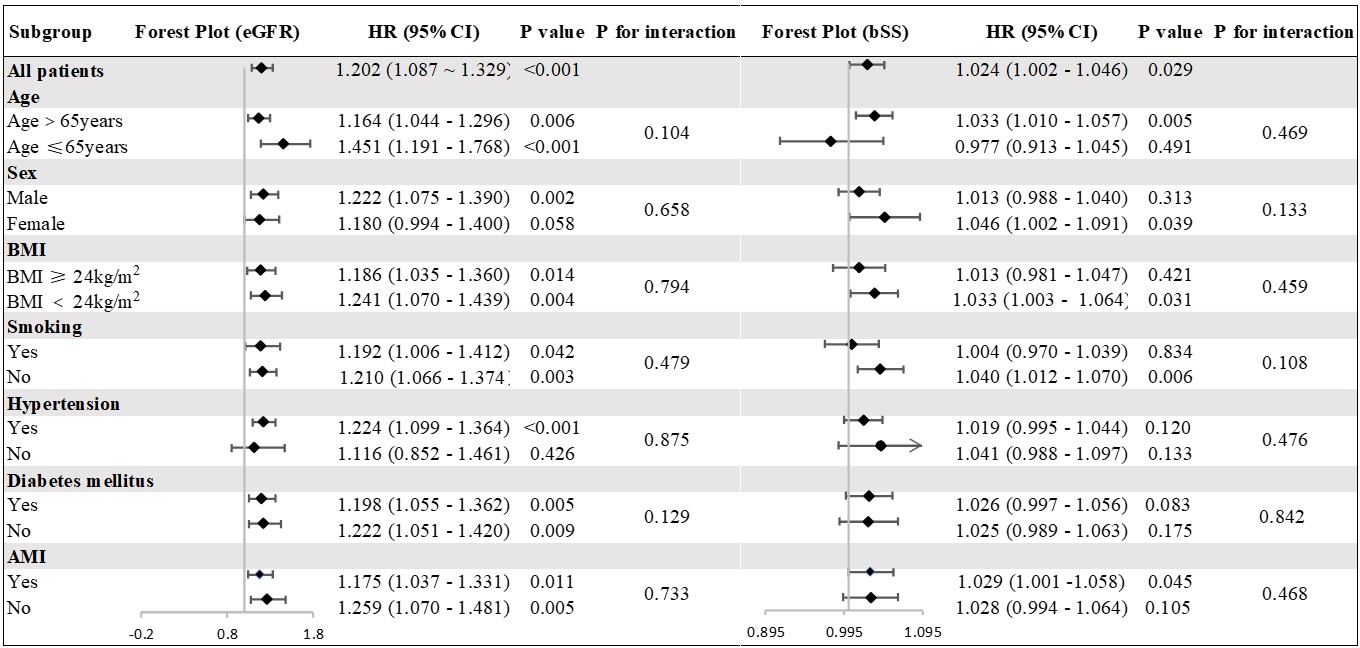


**Supplementary Fig. 9 Causal mediation analysis quantified bSS-mediated pathways in eGFR_Cr_-MACE associations across covariate-adjusted models.**

**
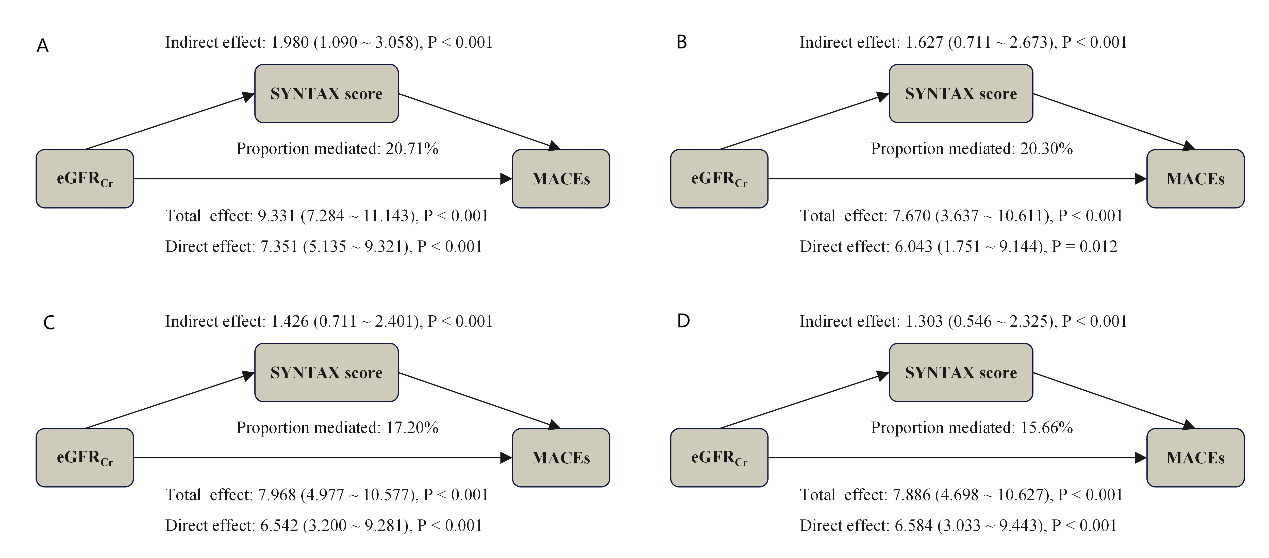
**

A represents the unadjusted Model; B represents the adjusted Model I; C represents the adjusted Model II; D represents the adjusted Model III.

Model I was adjusted for age, sex, BMI, HTN, DM, smoking, previous PCI, TG, TC, LDL-C, HDL-C;

Model II was adjusted for age, BMI, DM, Heart rate, Diuretics, Isu, aspirin, AMI, LVEF;

Model III was adjusted for Model I plus Model II.

**Abbreviations:** eGFR_Cr_, eGFR value derived solely from creatinine; PM, proportion mediated.

**Supplementary Fig. 10 Causal mediation analysis quantified bSS-mediated pathways in eGFR-Death associations across covariate-adjusted models.**

**
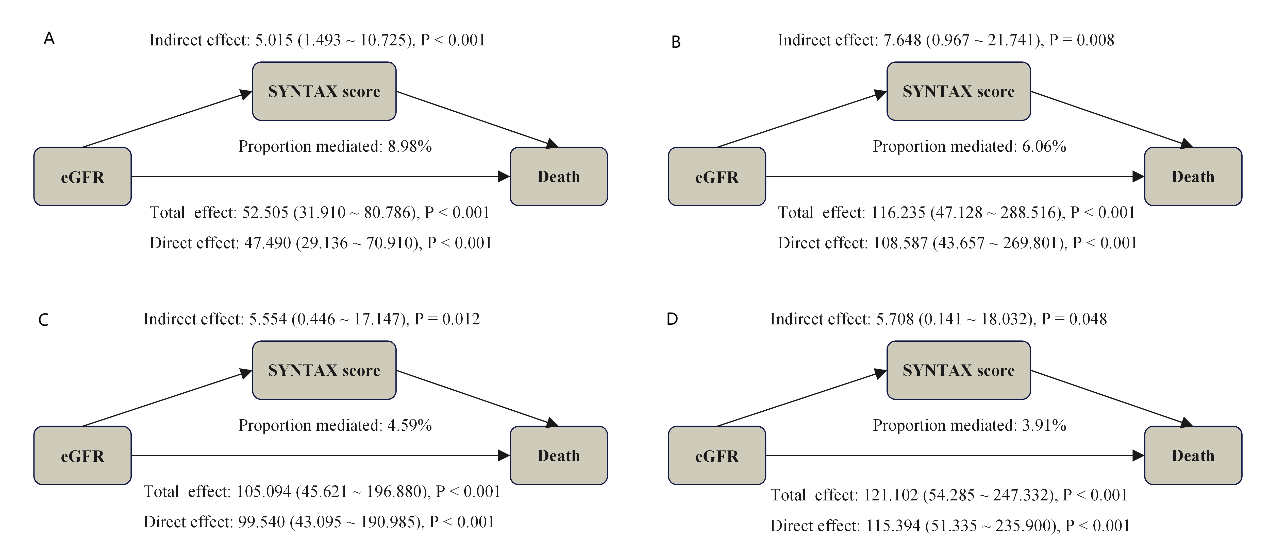
**

A represents the unadjusted Model; B represents the adjusted Model I; C represents the adjusted Model II; D represents the adjusted Model III.

Model I was adjusted for age, sex, BMI, HTN, DM, smoking, previous PCI, TG, TC, LDL-C, HDL-C;

Model II was adjusted for age, BMI, DM, Heart rate, Diuretics, Isu, aspirin, AMI, LVEF;

Model III was adjusted for Model I plus Model II.

**Abbreviations:** PM, proportion mediated.
